# Evaluating the Reliability of the Lesser Trochanter as a Landmark for Limb Length Discrepancy in Direct Anterior Approach Total Hip Arthroplasty

**DOI:** 10.7759/cureus.87418

**Published:** 2025-07-07

**Authors:** Supreet Bajwa, Ravi Teja Rudraraju, Kunal Aneja, Ponnanna Machaiah

**Affiliations:** 1 Department of Orthopaedics, Wockhardt Hospital, Mumbai Central, Mumbai, IND; 2 Department of Orthopaedics, Apollo Hospitals, Hyderabad, IND; 3 Department of Orthopaedics, Sri Venkata Sai (SVS) Medical College, Mahbubnagar, IND; 4 Department of Orthopaedics, Max Super Speciality Hospital, New Delhi, IND; 5 Department of Orthopaedics and Rehabilitation, Naveda Healthcare Centre, New Delhi, IND; 6 Department of Orthopaedics, Sparsh Hospital, Yeshwanthpur, Bengaluru, IND

**Keywords:** direct anterior approach, fluoroscopic validation, functional outcomes, lesser trochanter (lt), limb length discrepancy, total hip arthroplasty

## Abstract

Background and aim

Limb length discrepancy (LLD) is a common complication following total hip arthroplasty (THA), significantly impacting functional outcomes, patient satisfaction, and quality of life. The direct anterior approach (DAA) for THA has gained popularity due to its potential for minimizing LLD through precise intraoperative control. Despite advancements, achieving limb length equality remains challenging, particularly in the Indian patient population, where anatomical variations may affect surgical outcomes. The lesser trochanter (LT) is frequently utilized as a landmark for intraoperative LLD assessment. However, the reliability of the LT in DAA-THA remains debated. This study aimed to evaluate the accuracy and consistency of using the LT as an intraoperative reference for LLD correction in DAA-THA.

Methods

A retrospective cohort analysis was conducted on 130 patients who underwent DAA-THA at a high-volume tertiary care center between January 2023 and December 2023. Patients were selected based on the inclusion criteria of age >18 years, availability of preoperative and postoperative radiographs, and adequate fluoroscopic imaging during surgery. The LT was used as the primary landmark for limb length restoration. Intraoperative fluoroscopy and standardized leg positioning systems were employed to ensure accurate component placement. Preoperative and postoperative LLD were measured using standardized radiographic techniques, and functional outcomes were assessed through the Harris Hip Score (HHS), Western Ontario and McMaster Universities Osteoarthritis Index (WOMAC), and Forgotten Joint Score (FJS). Statistical analysis was performed to determine the association between LLD correction and functional recovery.

Results

The cohort had a mean age of 57.1 years, with 70% males and 30% females. The mean BMI was 27.1 ± 4.4 kg/m^2^. Primary indications were avascular necrosis (73.8%), femoral neck fractures (19.2%), rheumatoid arthritis (6.2%), and primary osteoarthritis (0.8%). The mean preoperative LLD of 1.5 cm was reduced to 0.2 cm postoperatively, with only two patients having LLD >1 cm.

Functional outcomes improved significantly postoperatively, with HHS increasing from 40.7 ± 5.7 preoperatively to 95.1 ± 4.4 at 12 months (p < 0.001). The WOMAC score decreased from 60.7 ± 5.8 to 10.1 ± 6.7 over the same period (p < 0.001). The FJS improved from 19.9 ± 6.45 preoperatively to 85.5 ± 9.3 postoperatively, indicating high patient satisfaction. Patients with postoperative LLD ≤0.5 cm had significantly higher HHS and lower WOMAC scores compared to those with residual LLD >1 cm, highlighting the importance of precise LLD correction for optimal functional recovery.

Conclusion

The LT serves as a reliable anatomical landmark for correcting LLD during DAA-THA, particularly when combined with intraoperative fluoroscopy and standardized positioning systems. This approach resulted in favorable postoperative functional outcomes and high patient satisfaction. Implementing standardized protocols that include LT-based measurements and fluoroscopic validation can significantly reduce LLD, enhancing clinical outcomes in THA. Further research is warranted to validate these findings in larger, multicenter cohorts.

## Introduction

Limb length discrepancy (LLD) following total hip arthroplasty (THA) continues to be a significant clinical challenge, with potential consequences including functional impairment, patient dissatisfaction, and increased risk of revision surgery [1,2]. Achieving precise limb length equality is a critical objective in THA, as even minor discrepancies can lead to gait abnormalities, lower back pain, and a decline in overall quality of life (QoL) [3]. The threshold for clinically acceptable LLD remains a subject of debate, with some studies suggesting that discrepancies of up to 10 mm may be tolerated, while others argue that even smaller differences can result in patient dissatisfaction [2].

The direct anterior approach (DAA) for THA has gained increasing popularity among orthopaedic surgeons due to its intermuscular nature, which is associated with several advantages, including reduced intraoperative bleeding, shorter hospital stays, and lower postoperative dislocation rates [2]. Additionally, the DAA facilitates the use of intraoperative fluoroscopy, enabling real-time assessment of component positioning, femoral offset, and LLD [4]. Despite these benefits, the superiority of the DAA in achieving consistent limb length restoration remains uncertain, necessitating further investigation into its reliability and effectiveness [3].

To minimize postoperative LLD, various methods have been proposed, which can be broadly categorized into preoperative templating, intraoperative clinical tests, navigation systems, and intraoperative measurements of anatomical landmarks [5]. Among the intraoperative measurements, the lesser trochanter (LT) is frequently utilized as a femoral reference point due to its accessibility and consistent anatomical location [6]. However, the reliability of the LT in ensuring accurate limb length restoration remains controversial, with limited evidence supporting its precision and reproducibility in the context of DAA [7].

The LT serves as a prominent and easily identifiable bony landmark on radiographs, making it a potential reference for intraoperative assessment of femoral length changes [8]. Nevertheless, its accuracy can be influenced by factors such as patient positioning, pelvic tilt, and radiographic magnification [9]. Furthermore, previous studies have reported inconsistent findings regarding LT’s reliability as a reference point for LLD measurement in THA, highlighting the need for further investigation [7].

Understanding the reliability of the LT in the context of DAA is crucial for optimizing surgical outcomes and minimizing the risk of LLD following THA. This study aims to evaluate the reliability of the LT as an intraoperative landmark for limb length restoration in patients undergoing THA via the DAA. Specifically, we compare preoperative and postoperative LLD using standardized anteroposterior (AP) pelvic radiographs to determine the radiological accuracy of the LT-based method. Additionally, we assess functional outcomes using validated clinical scores to examine the correlation between precise LLD correction and postoperative recovery.

## Materials and methods

Study design

A retrospective assessment of patient data retrieved from hospital records at a high-volume tertiary care center where DAA for THA is routinely performed between January 2023 and December 2023. A total of 154 patients were screened during the study period. Of these, 24 patients were excluded due to the following reasons: unavailability of adequate preoperative or postoperative radiographs (n = 11), incomplete intraoperative fluoroscopic documentation (n = 7), and a history of prior hip surgeries or congenital deformities (n = 6). Thus, the final analysis included 130 patients who met all inclusion criteria and had complete imaging and follow-up data. Data were extracted from electronic medical records, including operative notes and follow-up evaluations. Demographic variables such as age, sex, and preoperative body mass index (BMI) were systematically reviewed. Ethical approval was waived by the institutional review board due to the retrospective nature of the study. The research was conducted in accordance with the ethical principles of the Declaration of Helsinki, ensuring patient confidentiality and data integrity.

Patient demographics

The consecutive patients who met the inclusion criteria were enrolled in the study. Inclusion criteria comprised (i) patients aged >18 years with preoperative AP radiographs, (ii) patients with THA performed via DAA, (iii) availability of an adequate-quality intraoperative fluoroscopic image, and (iv) a standard postoperative pelvic radiograph. The fluoroscopic image was required to provide a centered view of the pelvis, ensuring visibility of both LTs and all final THA components, including the femoral head.

Exclusion criteria included a documented history of prior hip surgeries, congenital hip deformities, radiographic evidence of sub-trochanteric femoral deformities, and significant comorbidities contraindicating THA, such as advanced cardiovascular disease, uncontrolled diabetes, or severe renal dysfunction, as noted in preoperative evaluations. As this was a retrospective study, no direct patient contact occurred. Written informed consent for the use of anonymized clinical data in research was obtained preoperatively, ensuring adherence to ethical standards and the protection of patient confidentiality.

Surgical technique

All surgical procedures were conducted by an experienced orthopaedic surgeon adhering to a standardized DAA protocol. The technique prioritized minimal soft tissue disruption and precise implant positioning. Spinal or epidural anesthesia was uniformly administered, with no regional blocks utilized. Patients were positioned supine on a specialized fracture table, IOT Purist (IOT Orthopedics, Switzerland), equipped with a leg position traction system (LPTS). The operative limb was placed in neutral rotation with 10 degrees of abduction to facilitate femoral exposure and allow accurate length comparisons. The contralateral limb was kept in a similar extended position to maintain pelvic symmetry during fluoroscopic imaging.

Intraoperative fluoroscopy (C-arm) was employed after the final trial reduction to confirm implant positioning and to assess limb length equality. A standardized AP pelvic fluoroscopic view was obtained with the C-arm beam perpendicular to the table and centered over the symphysis pubis, ensuring both LTs were visible. Care was taken to match the projection with preoperative radiographs by minimizing parallax and keeping the pelvis square. Limb length was assessed by comparing the vertical height of the LT on the operative side to the contralateral LT using the ischial line or teardrop line as a reference [10]. LLD was recorded as a continuous variable with positive values indicating lengthening and negative values indicating shortening of the index limb relative to the contralateral side.

Intra-articular pericapsular injections of local anesthetics (ropivacaine or bupivacaine), ketorolac, and epinephrine were administered, with dosages adjusted according to individual body weight by the anesthesiologist. A single preoperative dose of antimicrobial prophylaxis was administered to all patients [10].

Implant selection and fixation methods were individualized based on surgeon preference and patient-specific factors, as outlined in prior literature [10].

LLD measurements

Preoperative and postoperative LLD were assessed using standardized AP pelvic radiographs, with the LT utilized as the primary anatomical reference point. Measurements were obtained from the most prominent aspect of the LT to a fixed pelvic reference line. Intraoperative evaluation was performed under fluoroscopic guidance, with adjustments implemented to achieve optimal limb length symmetry.

Outcome measures

Clinical evaluation of hip-related pain and functional outcomes was conducted using validated assessment tools, including the Harris Hip Score (HHS), the Western Ontario and McMaster Universities Osteoarthritis Index (WOMAC), and the Forgotten Joint Score (FJS). These assessments were performed at multiple time points: preoperatively, immediate postoperatively, at six weeks, and at 12 months post-surgery. Leg length discrepancy was measured both preoperatively and postoperatively, with a particular focus on patients exhibiting discrepancies exceeding 1 cm. Preoperative LLD was assessed using AP pelvic radiographs taken in an upright standing position. Similarly, postoperative AP pelvic radiographs were obtained in a standardized standing position at the time of discharge to ensure consistency in measurements.

Statistical analysis

Statistical analysis was conducted using IBM SPSS Statistics for Windows, Version 29 (Released 2022; IBM Corp., Armonk, New York, United States). Continuous variables were summarized as means ± standard deviations, while categorical variables were expressed as frequencies and percentages. Preoperative and postoperative LLD measurements were compared using a paired t-test. A p-value of <0.05 was considered statistically significant.

## Results

Patient demographics

This study comprised 130 patients with a mean age of 57.1 ± 19.2 years (range: 18-85 years), reflecting a demographic distribution encompassing both younger and older individuals. Males represented the majority of the study population (70%, n = 91), while females accounted for 30% (n = 39). The BMI of participants ranged from 20 to 35 kg/m^2^, with a mean BMI of 27.2 ± 4.4 kg/m^2^ (Table 1).

**Table 1:** Demographics characteristics of patients undergoing direct anterior approach total hip arthroplasty.

Variable	Mean ± SD/n (%)
No. of patients	130
Male	91 (70)
Female	39 (30)
Mean age (years)	57.14 ± 19.16
Mean body mass index (kg/m^2^)	27.14 ± 4.39

The primary etiology necessitating THA was avascular necrosis (AVN) (73.84%), consistent with the elevated prevalence of this condition in the Indian population. Femoral neck fractures (19.23%) were the second most common indication for THA, particularly prevalent among older patients. Rheumatoid arthritis (6.15%) and primary osteoarthritis (0.8%) were less frequently identified as underlying pathologies (Table 2).

**Table 2:** Primary indications of patients undergoing direct anterior approach total hip arthroplasty.

Primary diagnosis	N (%)
Avascular necrosis	96 (73.84)
Femoral neck fracture	25 (19.23)
Rheumatoid arthritis	8 (6.15)
Primary osteoarthritis	1 (0.76)

LLD measurements

In this study, the mean preoperative LLD was 1.5 ± 0.7 cm, indicating substantial limb asymmetry in the majority of patients prior to surgical intervention.

**Figure 1:**
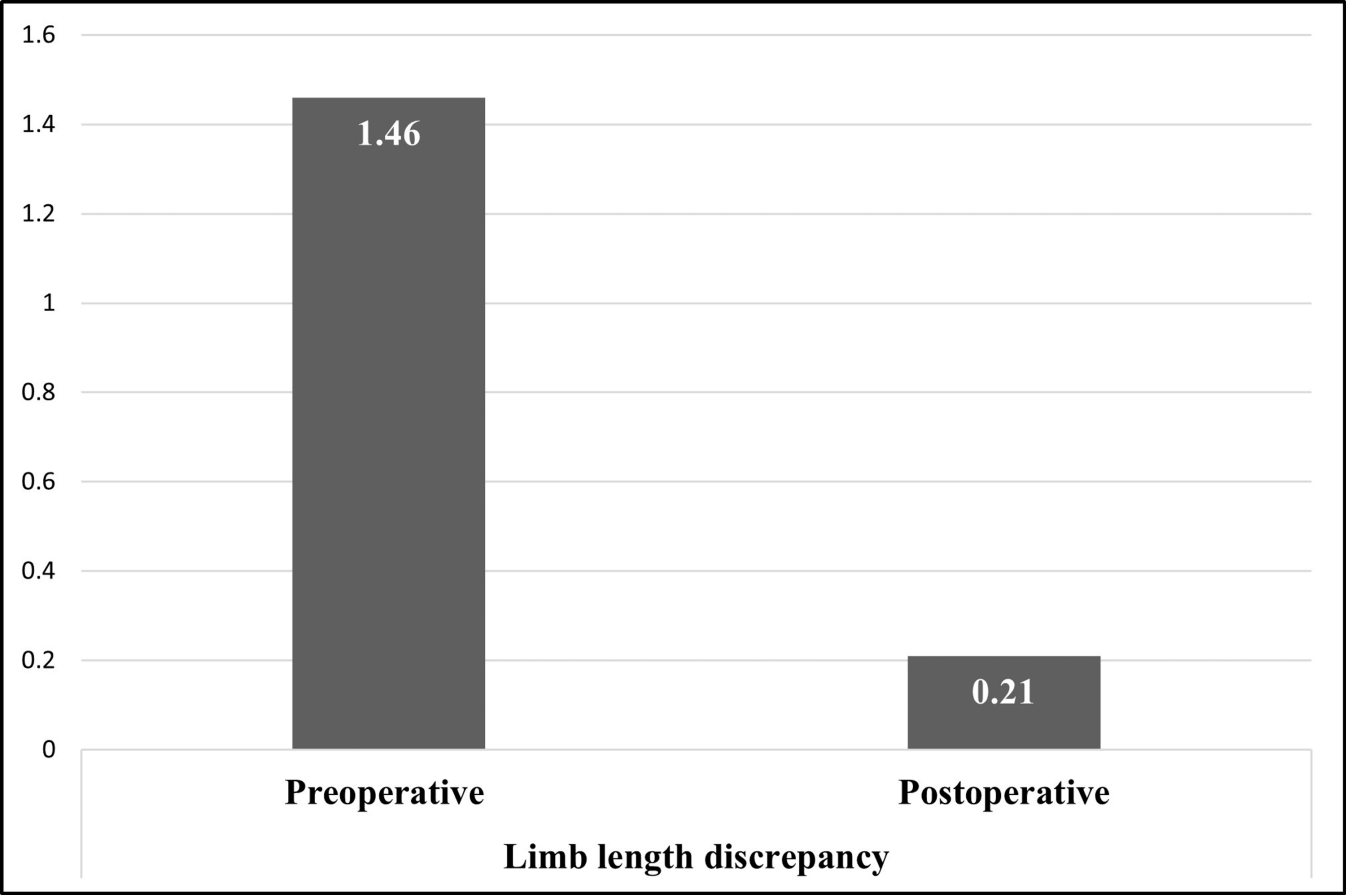
Limb length discrepancy after direct anterior approach total hip arthroplasty (in cm).

Following DAA-THA, a marked improvement in limb length symmetry was noted, with the mean postoperative LLD decreasing to 0.2 ± 0.2 cm (Figure 1).

**Figure 2:**
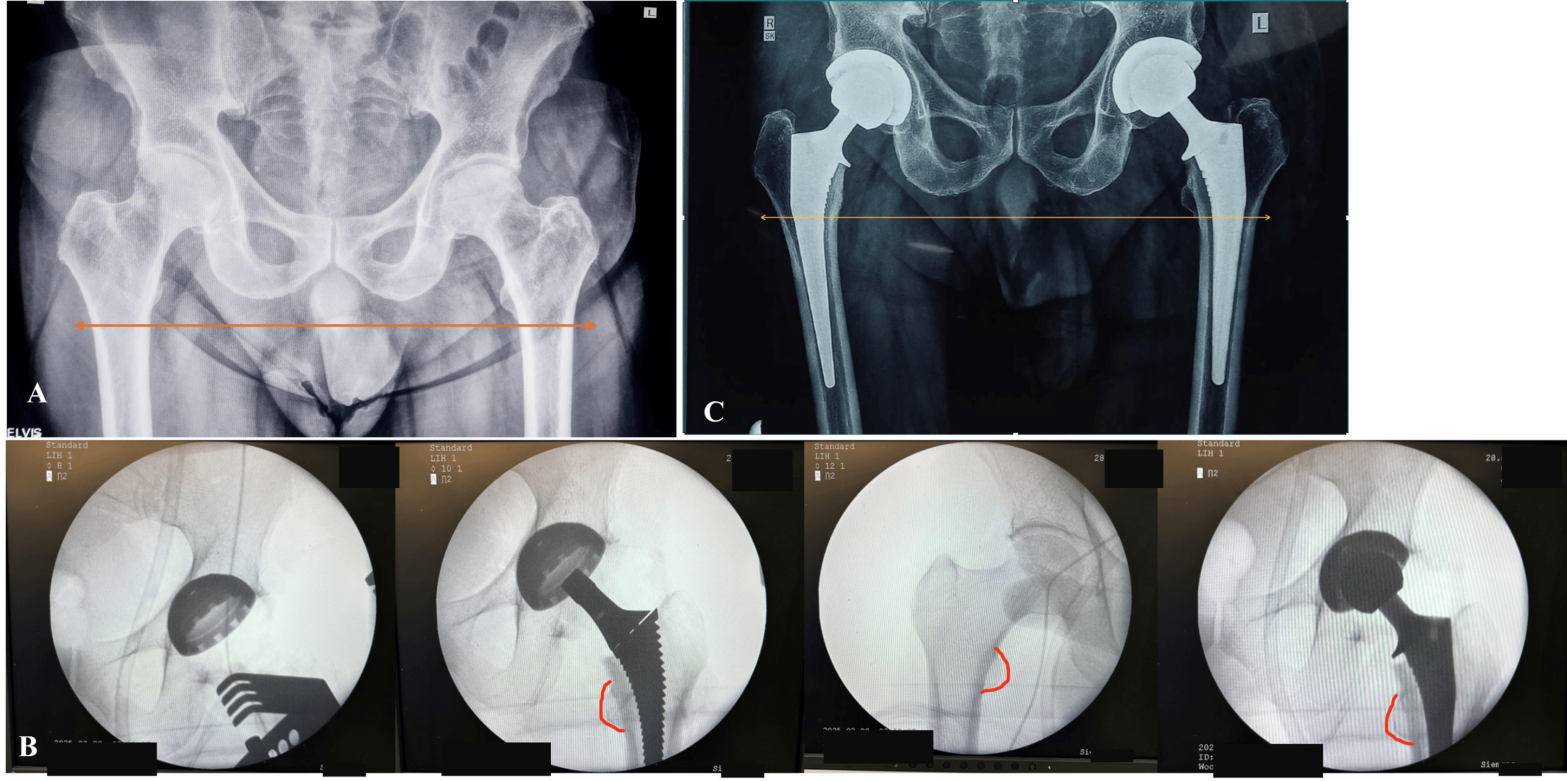
Radiographic assessment and intraoperative technique in direct anterior approach bilateral total hip arthroplasty (THA). Panel A: Preoperative radiograph of a 63-year-old patient with bilateral avascular necrosis (AVN) of the femoral head. Neither the patient nor the surgeon identified any actual or perceived limb length discrepancy (orange arrow). The patient underwent single-stage bilateral THA. Panel B: Intraoperative fluoroscopic images during left THA, utilizing the lesser trochanter (red curved lines) as an anatomical landmark for limb length assessment during a single-stage bilateral THA. Panel C: Immediate postoperative radiograph demonstrating the achieved leg length equality, confirming the accuracy of intraoperative limb length restoration (yellow arrow).

**Figure 3:**
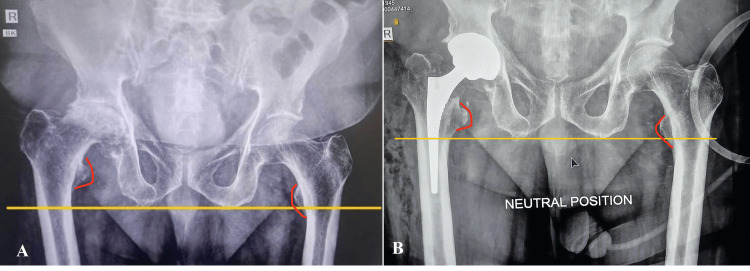
Radiographic evaluation of limb length discrepancy (LLD) following direct anterior approach total hip arthroplasty Panel A: Preoperative anteroposterior pelvic radiograph of a patient with significant limb length discrepancy (LLD, 2.6 cm) due to advanced hip pathology. The yellow reference line indicates the baseline limb length difference, and red curved lines show the difference in lesser trochanter position. Panel B: Immediate postoperative radiograph after total hip arthroplasty via the direct anterior approach showing partially corrected LLD to 1.5 cm postoperatively (yellow line) with the hip in a neutral position, with the lesser trochanter (red curved lines) as a point of reference.

Postoperatively, only two patients exhibited an LLD exceeding 1 cm. Notably, no patient demonstrated a postoperative LLD greater than 2 cm, reflecting the accuracy of the intraoperative assessment methods utilized (Figures 2-3).

Patients with postoperative LLD ≤ 0.5 cm exhibited significantly higher HHS and WOMAC scores compared to those with LLD > 0.5 cm (p < 0.05). These results underscore the importance of precise LLD correction in achieving optimal functional outcomes and improving patient satisfaction following THA.

Functional outcomes

Functional outcomes in this study were assessed using the HHS, WOMAC, and FJS scores. Patients without significant LLD showed excellent recovery, with notable improvements in HHS and reductions in WOMAC scores, indicating reduced pain and disability. Higher FJS scores in patients with minimal LLD suggested better joint awareness and satisfaction.

**Figure 4:**
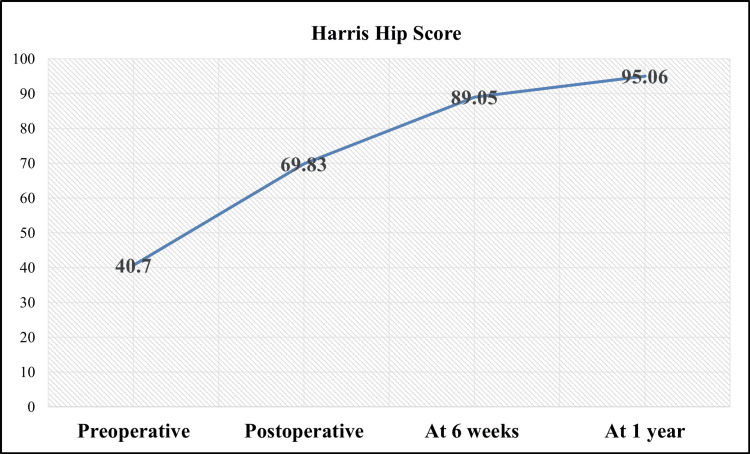
Harris Hip Score in patients undergoing direct anterior approach total hip arthroplasty.

Preoperatively, the mean HHS was 40.7 ± 5.7, reflecting substantial pain and functional impairment. The postoperative HHS improved to 69.8 ± 6.3, signifying early postoperative recovery. At six weeks, the score further increased to 89.1 ± 2.6, demonstrating substantial functional improvement, and by 12 months, it reached 95.1 ± 4.4, indicating excellent long-term outcomes (p < 0.001) (Figure 4).

**Figure 5:**
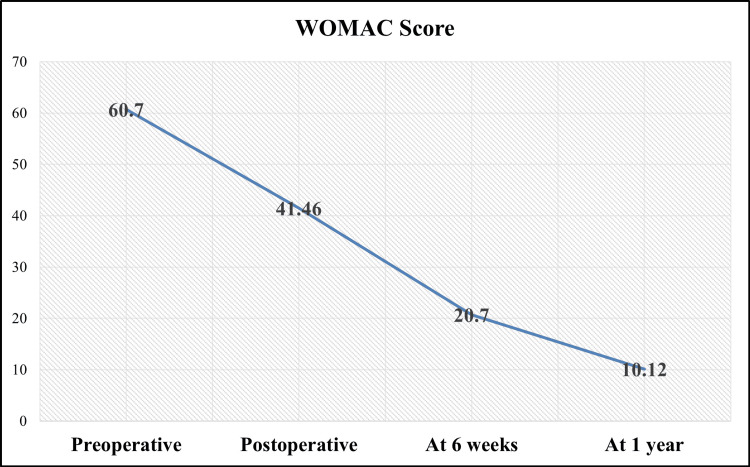
WOMAC scores in patients undergoing direct anterior approach total hip arthroplasty. WOMAC: Western Ontario and McMaster Universities Osteoarthritis Index

The WOMAC score, which evaluates pain, stiffness, and physical function, revealed a mean preoperative score of 60.7 ± 5.8, highlighting significant functional limitations. At discharge, the score decreased to 41.5 ± 5.2, reflecting initial postoperative improvements. By six weeks, it further declined to 20.7 ± 6.2, indicating continued pain relief and functional recovery. At 12 months, the WOMAC score was 10.1 ± 6.7, demonstrating substantial reductions in pain and stiffness, alongside enhanced physical function (p < 0.001), thereby underscoring the sustained benefits of DAA-THA (Figure 5).

**Figure 6:**
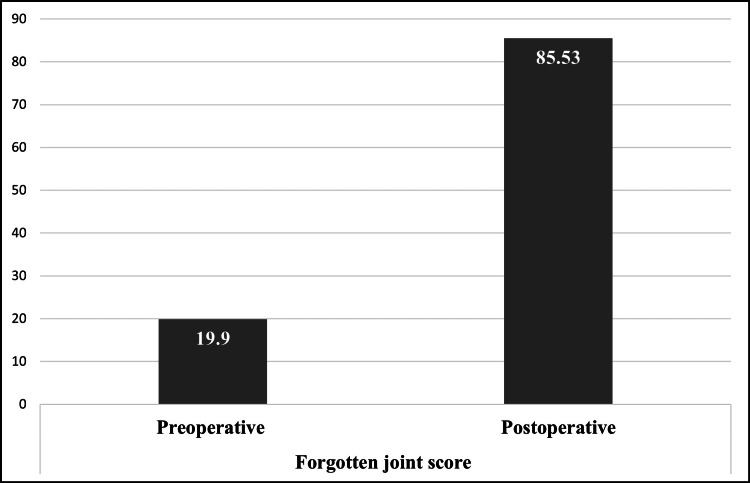
Forgotten joint score in patients undergoing direct anterior approach total hip arthroplasty.

The FJS evaluates patients' perception of their hip joint during routine daily activities. Preoperatively, the mean FJS was 19.9 ± 6.45, indicating substantial joint awareness and functional limitations. By the time of discharge, the mean FJS significantly improved to 85.5 ± 9.3, suggesting that patients had largely "forgotten" their hip joint during daily activities, indicative of high satisfaction levels and near-normal functional recovery. This improvement underscores the efficacy of DAA-THA in enhancing patients' QoL (Figure 6).

Case analysis of larger LLD

In this study, two cases with postoperative LLD exceeding 1 cm demonstrated lower HHS and WOMAC scores, alongside higher FJS, suggesting a perceived sense of joint unnaturalness despite functional limitations.

Patient 1, a 63-year-old male with a BMI of 34.3 kg/m^2^ (classified as obese class I), presented with a preoperative LLD of 2.6 cm, which was partially corrected to 1.5 cm postoperatively. Preoperative assessments revealed an HHS of 42 and a WOMAC score of 61, indicative of poor functional capacity and significant pain. Postoperatively, his HHS improved to 64 immediately, peaked at 80 by six weeks, and subsequently declined to 70 in one year. Concurrently, his WOMAC scores demonstrated substantial improvement, stabilizing at 35 by one year. The FJS increased markedly from 28 to 86, reflecting excellent prosthetic joint integration and high patient satisfaction. Nevertheless, the residual LLD and elevated BMI may have contributed to the observed decline in HHS at one year, potentially due to increased mechanical stress on the implant.

Patient 2, a 66-year-old male with a BMI of 28.4 kg/m^2^ (overweight), exhibited a preoperative LLD of 2.9 cm, which was corrected to 1.3 cm postoperatively. Preoperative HHS and WOMAC scores of 49 and 52, respectively, indicated significant functional impairment and pain. Postoperatively, his HHS improved to 80 immediately, reached 81 in six weeks, and subsequently declined to 72 in one year. WOMAC scores also showed significant improvement, stabilizing at 36 by one year. Similar to Patient 1, his FJS increased dramatically from 12 to 87, indicating excellent postoperative joint adaptation and high patient satisfaction.

Patient 2 achieved superior LLD correction compared to patient 1, which may account for his relatively more stable functional outcomes.

Both cases demonstrated notable improvements in pain relief, functional restoration, and joint awareness, underscoring the efficacy of THA in managing AVN. However, factors such as residual LLD, BMI, and potential mechanical stress on the implant may influence long-term outcomes, emphasizing the necessity for tailored postoperative care and monitoring.

## Discussion

The effectiveness of the LT as a landmark for managing LLD during DAA-THA presents significant clinical relevance. Our retrospective analysis reveals that using the LT allows for a systematic approach to reduce postoperative LLD, a common complication associated with varying degrees of patient dissatisfaction and functional impairment.

Assessment of LLD

Postoperative leg length equality is a critical objective in THA, as LLD is a prevalent complication, with reported incidences ranging from 1% to 27% in primary THA cases. The magnitude of LLD varies significantly, with discrepancies documented between 3 mm and 70 mm, and a mean range of 3-17 mm [5,11]. Clinically, LLD has been associated with adverse outcomes, including back pain, sciatica, neuritis, gait disturbances, patient dissatisfaction, prosthetic dislocation, and premature implant loosening [5,11]. Furthermore, LLD is a leading cause of litigation in orthopaedic surgery [5]. Traditionally, a discrepancy exceeding 10 mm has been deemed clinically significant, however, recent evidence suggests that gait abnormalities may occur with discrepancies as small as 5 mm [12]. Mitigating significant postoperative LLD remains a paramount challenge, as it is a primary source of patient dissatisfaction and legal disputes [13]. Achieving this goal necessitates the use of precise anatomical landmarks to guide surgical planning, intraoperative execution, and postoperative assessment [12].

Several strategies have been proposed to minimize postoperative LLD, including preoperative templating, intraoperative clinical tests, navigation systems, and intraoperative measurements of anatomical markers. Preoperative templating, particularly using pelvic radiographs, is a widely adopted approach [5]. A commonly utilized method involves drawing a line tangential to the inferior aspect of the ischial tuberosities and measuring the distance to the midpoint of the LT. This technique is less susceptible to distortion from pelvic positioning or implant placement, rendering it as a reproducible and reliable method [14].

Intraoperatively, the LT serves as a critical landmark due to its visibility and the attachment of the iliopsoas muscle. Its utility extends beyond surgical planning, providing a visual guide during the procedure [15]. However, the use of the LT version as a predictor of femoral anteversion remains contentious. While some studies highlight discrepancies in angles between the LT and femoral condyles or neck, others advocate for its use in preoperative templating, particularly when combined with intraoperative fluoroscopy [15]. Clinical investigation has demonstrated the effectiveness of incorporating the LT as a key anatomical landmark, particularly when combined with intraoperative fluoroscopy, in significantly reducing postoperative LLD [16]. In our study, the mean LLD was reduced from 1.5 cm preoperatively to 0.2 cm postoperatively, accompanied by high levels of patient satisfaction. These results are consistent with the findings of Ishii et al., who reported enhanced LLD correction using LT-based measurements in THA performed via DAA. Their comparative analysis of dual mobility and single mobility implants revealed no statistically significant differences in LLD outcomes between the two groups, indicating that the technique's efficacy is independent of implant design [16].

Prospective studies have reported favorable outcomes, with 88.2% of patients exhibiting postoperative LLD of less than 5 mm and 94.1% having LLD under 10 mm, with an average discrepancy of 2.5 mm [5]. Similarly, retrospective analyses have shown that 96% of patients achieved LLD ≤ 10 mm and 77% achieved LLD ≤ 5 mm, indicating high levels of postoperative satisfaction [13]. However, a recent study cautions that a significant proportion of LLD may occur distal to the LT, underscoring the need for comprehensive assessment methods [17]. A study utilizing full-length standing AP radiographs found that approximately one-sixth of patients exhibited an LLD >10 mm when measured from the LT [18]. Similarly, a retrospective analysis of 100 patients reported that 15% exhibited an LLD >10 mm when measured from the LT to the talus [17].

Comparative studies between surgical approaches, such as the DAA and posterior approach, indicate that DAA is associated with a lower incidence of LLD > 10 mm (1.2% vs. 3.7%, respectively) [2]. The reduced LLD observed with the anterior approach, as corroborated by our study, may be attributed to the supine position employed during the procedure. This positioning enhances the utility of intraoperative fluoroscopy enabling more precise leg-to-leg comparisons [19]. These results underscore the critical role of accurate intraoperative techniques in reducing LLD. Compared to traditional clinical assessments, which rely on subjective judgment, intraoperative measurement techniques offer a more objective and standardized approach to ensuring limb length equality.

Intraoperative techniques

Intraoperative fluoroscopy has emerged as a reliable and cost-effective method for real-time assessment of leg length during THA via the anterior minimally invasive surgical approach, obviating the need for soft tissue tension tests [5]. This method is particularly beneficial in scenarios where direct leg measurements are unfeasible, such as when patients are secured on a traction table [2]. The strategic application of fluoroscopy in the DAA enhances the precision of component placement and leg length equalization, as evidenced by its utility in on-table procedures [10]. However, preoperative planning methods, such as digital templating, are constrained by inconsistencies in patient positioning, which can compromise their intraoperative reproducibility [2,5].

To mitigate LLD intraoperatively, it is critical to establish stable anatomical reference points in both the pelvis and femur as well as, to accurately replicate the femur's abduction/adduction orientation before and after trial component placement [5]. Evidence suggests that fluoroscopy during DAA significantly reduces postoperative LLD compared to non-fluoroscopic posterior approaches. For instance, a study compared the utilization of fluoroscopy in DAA-THA with cases employing the posterior approach without fluoroscopy. The study revealed a statistically significant reduction in postoperative LLD when fluoroscopy was employed, with mean discrepancies of 0.7 mm in the fluoroscopy group compared to 2.7 mm in the non-fluoroscopy group [20]. Similarly, a retrospective study measured LLD using a single intraoperative fluoroscopic image and reported an LLD of 2.4 ± 2.1 mm, with 1.2% of patients exhibiting LLD > 10 mm [4].

Similarly, Blum et al. demonstrated that fluoroscopy in the supine position during the anterior-based muscle-sparing approach resulted in fewer cases of LLD > 5 mm compared to non-fluoroscopic lateral positioning, and fewer instances of LLD > 10 mm relative to posterior approaches without fluoroscopy [21]. A single‑center study of 244 consecutive hips by the experienced surgeon found that DAA yielded a median LLD of 0 mm, significantly better than the posterolateral approach, which had a median LLD of 5 mm (p < 0.0001) [22]. However, conflicting findings have been reported by Bingham et al., who observed no significant difference in postoperative LLD with or without fluoroscopy when DAA-THA was performed by highly experienced surgeons, achieving mean LLDs of 1.1 mm and 0.8 mm, respectively [23].

Fluoroscopy also enhances implant positioning accuracy. A retrospective study of 160 patients demonstrated superior cup positioning in DAA cases with fluoroscopic assistance compared to posterior approaches [24]. Despite these benefits, fluoroscopy is not without limitations, including radiation exposure, prolonged surgical time, potential image distortion due to parallax [3,4], and significant cost [4]. Nonetheless, its role in intraoperative assessment of leg length, offset, component size, and implant positioning remains invaluable [4].

Accurate assessment of LLD intraoperatively is highly dependent on proper limb positioning and radiographic alignment. Even minor changes in hip rotation, abduction, or flexion can significantly alter the projected position of the LT on fluoroscopic or radiographic images. Internal rotation tends to obscure the LT and shorten its visible prominence, while external rotation enhances its projection, potentially leading to perceived discrepancies in leg length. Similarly, limb abduction or adduction can alter pelvic tilt and parallax distortion, affecting vertical alignment.

In our technique, careful attention was paid to limb alignment using the LPTS table to standardize positioning during fluoroscopy. Matching limb position with preoperative imaging and ensuring neutral pelvic orientation was essential to minimize measurement error. These considerations are critical to achieving true limb symmetry and preventing misinterpretation of intraoperative imaging when using the LT as a reference.

Recent advancements, such as the use of external grids and digital overlay navigation, have further refined outcomes. The systematic review highlighted that the use of fluoroscopy combined with external grids resulted in an average postoperative LLD of 2.9 ± 2.2 mm, while real-time fluoroscopic navigation demonstrated a notable reduction in LLD from 4.5 ± 3.2 mm to 1 ± 1.2 mm [2]. Additionally, the integration of leg positioning systems, such as the IOT Purist, has minimized variability in patient positioning, further reducing the risk of unintentional LLD [10].

The findings of this study underscore the efficacy of intraoperative fluoroscopy, particularly when utilized in conjunction with the LT as a stable anatomical reference point. The integration of the IOT Purist leg positioning system in this study significantly reduced variability in patient positioning, thereby mitigating the risk of unintentional LLD attributable to intraoperative malpositioning. Additionally, the LPTS enhances proximal femoral access and delivery, addressing challenges such as inadequate femoral exposure, which can contribute to complications, particularly during the initial stages of mastering this surgical technique [10].

Functional outcomes and LLD

Our findings indicate that patients with minimal LLD following DAA-THA demonstrated significantly better HHS and lower WOMAC scores, reflecting improved functional recovery and reduced pain levels. These results are consistent with prior observations, where HHS and WOMAC scores showed progressive improvement from the preoperative period to the third postoperative month and continued to improve thereafter. Notably, no significant correlation was observed between changes in LLD and improvements in HHS or WOMAC scores over time [25]. However, in the two cases from our study with LLD exceeding 1 cm, lower HHS and higher FJS were recorded, suggesting increased awareness of the operated joint and potential compensatory gait adaptations. However, this observation remains descriptive and should be interpreted with caution due to the small subgroup size. Among these, one patient had a lengthening and the other a shortening, underscoring the variability in individual tolerance and perception.

These findings are consistent with previous studies, which have reported that patients with perceived LLD tend to experience poorer clinical outcomes, including lower Oxford Hip Scores (OHS) and reduced satisfaction [26,27]. Notably, a prospective non-randomized study found that patients with LLD ≥ 10 mm had significantly worse OHS at one and three years postoperatively [26]. Similarly, another study highlighted the functional impact of patient-perceived LLD, noting its association with poorer outcomes and increased limping frequency [27].

Restoration of LLD remains a critical objective in THA, as discrepancies have been linked to complications such as sciatic nerve palsy, gait abnormalities, and chronic pain [11]. Additionally, perceived LLD has been associated with diminished functional outcomes and reduced patient satisfaction. For instance, Iversen et al. reported that patients who perceived LLD had median HHS and satisfaction scores of 83 and 92, respectively, compared to 92 and 100 in those without perceived LLD [28]. Similarly, Konyves and Bannister observed a decreased OHS at 3 and 12 months postoperatively in patients with perceived LLD [29], while Wylde et al. noted an association between perceived LLD and diminished OHS at mid-term follow-up [27].

A comparative study has highlighted the advantages of the DAA over the posterior lateral approach in THA, with the DAA group achieving higher HHS at six weeks postoperatively, indicative of faster functional recovery and earlier return to daily activities [19]. However, no significant differences in HHS were observed between the two approaches at one- and five-year follow-ups. Furthermore, while a weak negative correlation between LLD and HHS was noted at six weeks in cases with LLD > 0, no such correlation was evident at longer-term follow-ups [19]. These findings suggest that while LLD may influence short-term outcomes, its impact on long-term functional recovery may be limited.

Despite these findings, some studies have reported no significant differences in clinical outcomes, such as HHS, 12-Item Short Form Survey (SF-12), or Postel-Merle d’Aubigné scores, between patients with and without postoperative LLD, potentially due to limited statistical power [1]. This underscores the need for further research to elucidate the relationship between LLD and functional outcomes, particularly in the context of surgical approach and patient perception. Overall, while LLD appears to influence short-term recovery and patient satisfaction, its long-term impact on functional outcomes remains less clear, warranting continued investigation.

Implications for clinical practice

The findings of this study underscore the clinical utility of the LT as a dependable intraoperative landmark for minimizing LLD in THA performed via DAA. The LT’s visibility during surgery is facilitated by its anatomical attachment to the iliopsoas muscle on its anterior surface, making it a reliable reference point. This bony landmark serves a dual purpose: it aids in preoperative planning and acts as a visual guide during surgery for estimating femoral anteversion [15]. Owing to its accessibility and reproducibility, LT provides a practical and cost-effective solution for improving intraoperative accuracy without the need for advanced technologies.

Findings from our study also advocate for the implementation of standardized intraoperative protocols to improve surgical precision, including routine fluoroscopic validation to ensure the accurate positioning of the LT and achieve limb length symmetry, as well as the use of standardized positioning systems such as the IOT Purist LPTS to minimize procedural inconsistencies. The LPTS has been shown to facilitate optimal proximal femoral exposure while reducing complications such as calcar fractures, canal perforation, and greater trochanteric fractures [10]. Adoption of the on-table DAA may improve visualization and facilitate precise anatomical alignment, potentially decreasing the risk of intraoperative fractures and nerve injuries. The inherent standardization of this approach enables more accurate fluoroscopic-assisted component placement, contributing to improved limb length equalization and a reduced learning curve for surgical teams [10]. Lastly, training and incorporation of landmark-based techniques in surgical education may enhance consistency in LLD correction and overall procedural accuracy.

These strategies collectively reinforce the importance of structured, reproducible methodologies in THA to optimize patient outcomes while ensuring intraoperative precision.

Limitations

Despite the promising findings of this study, several limitations must be acknowledged. The retrospective design inherently limits the ability to establish causal relationships between the use of the LT as a reference point and postoperative LLD outcomes. Prospective studies with larger sample sizes are needed to validate these findings. The reliance on intraoperative fluoroscopy and standardized positioning systems, such as the IOT Purist leg positioning system, may not be feasible in all surgical environments due to cost and resource constraints. The study did not account for potential confounding factors such as variations in pelvic tilt, femoral anatomy, or patient-specific biomechanics, which could influence the accuracy of LLD measurements. While our findings and supporting literature suggest that intraoperative fluoroscopy with LT-based referencing yields superior or comparable outcomes to posterior or lateral approaches without imaging, this study did not include a direct comparison across different surgical approaches. Therefore, the reproducibility of this technique in lateral or posterior approaches remains unverified. All intraoperative measurements were performed by a single experienced surgeon, which may introduce operator-dependent bias. The reproducibility of the LT-based technique across different surgeons or institutions remains to be validated in multicenter or prospective trials. The follow-up period was limited to 12 months, which may not capture long-term functional outcomes or complications associated with LLD.

## Conclusions

This study emphasizes the clinical significance of the LT as a dependable anatomical landmark for reducing LLD in THA conducted via DAA. The findings reveal that the LT, when utilized alongside intraoperative fluoroscopy and standardized surgical protocols, serves as a consistent and reproducible reference for minimizing postoperative LLD. The mean postoperative LLD was reduced to 0.2 cm, with only two cases exceeding 1 cm, demonstrating the precision of this methodology. Additionally, patients with minimal LLD achieved significantly superior functional outcomes, as evidenced by improvements in the HHS, WOMAC, and FJS, highlighting the critical role of precise limb length restoration in optimizing clinical results.

The implementation of standardized protocols, such as routine fluoroscopic validation, the use of advanced leg positioning systems, and the integration of landmark-based techniques into surgical training, can significantly enhance the accuracy and reproducibility of LLD correction. While the DAA offers distinct advantages in reducing LLD and facilitating early functional recovery, further research is warranted to investigate the long-term implications of LLD on patient outcomes and to refine intraoperative assessment techniques. These advancements will contribute to the continued evolution of THA, ultimately improving surgical precision and patient care.

## References

[1] Lecoanet P, Vargas M, Pallaro J, Thelen T, Ribes C, Fabre T (2018). Leg length discrepancy after total hip arthroplasty: can leg length be satisfactorily controlled via anterior approach without a traction table? Evaluation in 56 patients with EOS 3D. Orthop Traumatol Surg Res.

[2] Tassinari L, Di Martino A, Brunello M, Rossomando V, Traina F, Faldini C (2024). Leg length discrepancy after total hip arthroplasty performed by direct anterior approach: a systematic review comparing surgical approaches and strategies for prevention. EFORT Open Rev.

[3] O'Leary R, Saxena A, Arguelles W, Hernandez Y, Osondu CU, Suarez JC (2022). Digital fluoroscopic navigation for limb length restoration during anterior total hip arthroplasty. Arthroplast Today.

[4] Caus S, Reist H, Bernard C, Blankstein M, Nelms NJ (2021). Reliability of a simple fluoroscopic image to assess leg length discrepancy during direct anterior approach total hip arthroplasty. World J Orthop.

[5] Girolami M, Bevoni R, Artioli E (2024). An intraoperative method to minimize leg length discrepancy in anterior minimally invasive total hip arthroplasty-a prospective study. J Pers Med.

[6] Marchand LS, Todd DC, Kellam P, Adeyemi TF, Rothberg DL, Maak TG (2018). Is the lesser trochanter profile a reliable means of restoring anatomic rotation after femur fracture fixation?. Clin Orthop Relat Res.

[7] Kim JI, Moon NH, Shin WC, Suh KT, Jeong JY (2017). Reliable anatomical landmarks for minimizing leg-length discrepancy during hip arthroplasty using the lateral transgluteal approach for femoral neck fracture. Injury.

[8] Hasler J, Hoch A, Fürnstahl P, Ackermann J, Zingg PO, Vlachopoulos L (2021). Is the contralateral lesser trochanter a reliable reference for planning of total hip arthroplasty - a 3-dimensional analysis. BMC Musculoskelet Disord.

[9] Hamad MN, Livshetz I, Sood A, Patetta M, Gonzalez MH, Amirouche FA (2022). Effects of pelvic obliquity and limb position on radiographic leg length discrepancy measurement: a Sawbones model. J Exp Orthop.

[10] Bajwa S (2023). Surgical technique of direct anterior approach for primary total hip arthroplasty using a leg positioning traction system. J Orthop Case Rep.

[11] Desai AS, Dramis A, Board TN (2013). Leg length discrepancy after total hip arthroplasty: a review of literature. Curr Rev Musculoskelet Med.

[12] Vermuyten L, Driesen R, Welters H, Corten K (2022). The obturator externus as surgical landmark for the direct anterior approach and its role in LLD after total hip replacement. J Arthrosc Jt Surg.

[13] Doehrmann R, Comer BJ, Chatterji R, Diedring B, Knapp P, Afsari A (2023). Accuracy of leg length and hip offset measurements using a fluoroscopic grid during anterior approach total hip arthroplasty. Arthroplast Today.

[14] Heaver C, St Mart JP, Nightingale P, Sinha A, Davis ET (2013). Measuring limb length discrepancy using pelvic radiographs: the most reproducible method. Hip Int.

[15] Mohamed Thajudeen MZb, Mahmood Merican A, Hashim MS, Nordin A (2022). The utility of lesser trochanter version to estimate femoral anteversion in total hip arthroplasty: a three-dimensional computed tomography study. Surg Tech Dev.

[16] Ishii S, Homma Y, Baba T (2021). Does total hip arthroplasty via the direct anterior approach using dual mobility increase leg length discrepancy compared with single mobility?. Arthroplasty.

[17] Gallo MC, Chung BC, Tucker DW, Piple AS, Christ AB, Lieberman JR, Heckmann ND (2021). Limb length discrepancy in total hip arthroplasty: is the lesser trochanter a reliable measure of leg length?. J Arthroplasty.

[18] Wu J, Zhuang X, Lin C, He L, Zhang R (2023). Does the use of intraoperative measurement reduce limb length discrepancies after total hip arthroplasty?. BMC Musculoskelet Disord.

[19] Lu Y, Li X, Hu Y, Zhao F (2025). Comparison of leg length discrepancy after total hip arthroplasty: direct anterior and posterior lateral approach. PLoS One.

[20] Leucht P, Huddleston HG, Bellino MJ, Huddleston JI (2015). Does intraoperative fluoroscopy optimize limb length and the precision of acetabular positioning in primary THA?. Orthopedics.

[21] Blum CL, Luzzi AJ, Frederick JS (2024). Intraoperative fluoroscopy decreases magnitude and incidence of leg-length discrepancy following total hip arthroplasty. Arthroplast Today.

[22] Wu PK, Chang WS, Chen KT, Huang PC, Ho CH, Chien CS, Wu TM (2024). Does the utilization of fluoroscopy affect the accuracy of prosthesis position in patients undergoing hip replacement surgery via the direct anterior approach compared to the posterolateral approach for an experienced surgeon? A single-center retrospective study. BMC Musculoskelet Disord.

[23] Bingham JS, Spangehl MJ, Hines JT, Taunton MJ, Schwartz AJ (2018). Does intraoperative fluoroscopy improve limb-length discrepancy and acetabular component positioning during direct anterior total hip arthroplasty?. J Arthroplasty.

[24] Kobayashi H, Homma Y, Baba T, Ochi H, Matsumoto M, Yuasa T, Kaneko K (2016). Surgeons changing the approach for total hip arthroplasty from posterior to direct anterior with fluoroscopy should consider potential excessive cup anteversion and flexion implantation of the stem in their early experience. Int Orthop.

[25] Smolle MA, Fischerauer SF, Maier M (2021). Leg length measures appear inaccurate in the early phase following total hip arthroplasty. Sci Rep.

[26] Beard D, Palan J, Andrew J, Nolan J, Murray DW (2008). Incidence and effect of leg length discrepancy following total hip arthroplasty. Physiotherapy.

[27] Wylde V, Whitehouse SL, Taylor AH, Pattison GT, Bannister GC, Blom AW (2009). Prevalence and functional impact of patient-perceived leg length discrepancy after hip replacement. Int Orthop.

[28] Iversen MD, Chudasama N, Losina E, Katz JN (2011). Influence of self-reported limb length discrepancy on function and satisfaction 6 years after total hip replacement. J Geriatr Phys Ther.

[29] Konyves A, Bannister GC (2005). The importance of leg length discrepancy after total hip arthroplasty. J Bone Joint Surg Br.

